# Mg-Al Mixed Oxide Adsorbent Synthesized Using FCT Template for Fluoride Removal from Drinking Water

**DOI:** 10.1155/2019/5840205

**Published:** 2019-07-07

**Authors:** Jifa Liu, Ping Zhao, Yue Xu, Xibin Jia

**Affiliations:** Key Laboratory of Processing and Testing Technology of Glass & Functional Ceramics of Shandong Provincial, School of Materials Science and Engineer, Qilu University of Technology (Shandong Academy of Sciences), Jinan 250353, China

## Abstract

To make full use of natural waste, a novel Mg-Al mixed oxide adsorbent was synthesized by the dip-calcination method using the fluff of the chinar tree (FCT) and an Mg(II) and Al(III) chloride solution as raw materials. The adsorbents were characterized by X-ray diffraction (XRD), scanning electron microscopy (SEM), Fourier transform infrared (FT-IR) spectroscopy, and X-ray photoelectron spectroscopy (XPS). The effects of the Mg/Al molar ratio and calcination temperature on the performance of the novel Mg-Al mixed oxide adsorbent were investigated. The optimized Mg-Al mixed oxide adsorbent had a Langmuir adsorption capacity of 53 mg/g. This adsorption capacity was higher than that of the separate Mg oxide and Al oxide. The synergy between Mg and Al is beneficial to the adsorption performance of the material. The fluoride adsorption capacity of the optimized Mg-Al mixed oxide adsorbent is only slightly affected by ions such as Cl^−^, NO_3_^−^, SO_4_^2−^, Na^+^, and K^+^ and is excellent for use in recycling and real water. The hydroxyl groups on the surface of the Mg-Al mixed oxide adsorbent play a key role in the adsorption of fluorine. The as-obtained novel Mg-Al mixed oxide adsorbent is an efficient and environmentally friendly agent for fluoride removal from drinking water.

## 1. Introduction

Microstructure plays a vital role in determining the performance of materials. In nature, plants and organisms have formed a large number of unique microstructures through long-term evolution and natural selection. The biological template of nature provides new directions for high-performance and multifunctional materials [1]. In recent years, researchers have used biological templates to prepare a large number of high-performance materials [2]. To date, biological materials, such as spherical yeast [3], viruses [4], cotton cellulose [5], nucleotides [6], and poplar catkin [7], have been used as templates. Combining biomaterials and chemical materials is one way to use natural resources to serve humanity.

With the development of human civilization, the pollution of water resources has emerged as a main issue requiring an urgent solution. In addition, high-fluoride drinking water is harmful to human health, and the fluoride content of drinking water has exceeded the WHO standard of 1.5 mg/L in some countries [8, 9]. Long-term drinking of water with excess fluoride can lead to abnormal tooth enamel in children and to joint pain and deformity of the limbs in adults [10]. Various methods of fluoride removal have their own limitations, but the adsorption method is regarded as the most promising method because of its simple process and low cost [11, 12]. At the same time, the adsorption method is also widely used to remove other pollutants such as chromium (IV) [13, 14]. To date, Ti(IV)-modified granular activated carbon [15], cerium-containing bone char [16], activated alumina [17], Mn-Ce oxide [18], Al-Fe (hydr)oxides [19], a magnesium-iron-aluminum trimetal composite [20], and hydrous zirconium oxide [21] have been used as fluoride adsorbents. The addition of precious metals and an increase in the complexity of the preparation techniques both increase the cost of the adsorbent, which is one of the factors hindering the use of adsorbents in developing countries.

In recent years, activated alumina and magnesium oxides have been used widely due to their mesoporous structure, low toxicity, recyclability, and modifiability [22–27]. Sabu et al. [28] used egg shell membrane as a template to synthesize hierarchical interwoven alumina as a fluoride adsorbent. Zhang et al. [29] prepared hierarchical microstructured/nanostructured tubular TiO_2_ using the fluff of the chinar tree (FCT) as a biological template. The resultant material is used as a photocatalyst, and its catalytic performance is enhanced by its porous and tubular structure. In addition, FCT can easily cause skin irritation and respiratory infections. Using FCT as a biological template not only reduces its harm but also makes full use of natural resources to serve humanity. However, the literature contains few reports on the use of FCT as a biological template for the preparation of a fluoride adsorbent.

In this paper, Mg-Al mixed oxide hollow tubes were synthesized using the dip-calcination method with FCT as a biological template and with aluminum chloride and magnesium chloride as precursors without adding any precipitant. The fluoride adsorption and synergistic interactions between Mg and Al during crystallization were investigated. The adsorption mechanism of the Mg-Al bimetal mixed oxide adsorbent is discussed.

## 2. Experimental

### 2.1. Materials and Methods

FCT was collected from the campus of our university. Aluminum chloride, magnesium chloride, and sodium fluoride were supplied by Sinopharm Chemical Reagent Co., Ltd. The FCT was washed with deionized water after being dipped in anhydrous ethanol and 0.1 M hydrochloric acid and was dried in an electrical drying oven at 60°C. Oxide adsorbent powders were prepared via a dip-calcination method. The FCT was immersed in 0.3 mol/L water solution of AlCl_3_·6H_2_O and MgCl_2_·6H_2_O for 24 h at room temperature. It was then dried in an electrical drying oven at 60°C. The FCT loaded with aluminum chloride and magnesium chloride was placed in a corundum crucible and calcined at 400, 600, 800, or 1000°C in a muffle furnace for 120 min individually. The adsorbent adsorption capacity was optimized by adjusting the calcination temperature and Mg/Al molar ratio.

### 2.2. Adsorbent Characterization

An Autosorb-iQ-MP surface area and pore size analyzer (Quantachrome Instruments) were used to record the Brunaurer–Emmett–Teller (BET) data of prepared oxide adsorbent powders at 77.35 K using nitrogen. The phase structure of the prepared oxide adsorbent powder was characterized by CuK_α_ radiation with a wavelength of *λ* = 0.15418 nm on a SHIMADZU XRD-6100 X-ray diffractometer. The X-ray diffractometer scan range was 10–70°, the speed was 6°/min, and the scanning step was 0.02°. Scanning electron microscopy (SEM) images of the oxide adsorbent powder were obtained from a ZEISS GeminiSEM 500 field emission scanning electron microscope. The Fourier transform infrared (FT-IR) spectra of the oxide adsorbent powders before and after adsorption were recorded in the range 4000–500 cm^−1^ using a Thermo Scientific Nicolet iS10 FT-IR spectrometer. X-ray photoelectron spectroscopy (XPS) analysis of oxide adsorbent powders before and after adsorption was conducted on a Thermo Scientific Escalab 250Xi spectrometer using an Al *K*_*α*_ X-ray source (1486.6 eV, 150 W) as a constant analyzer.

### 2.3. Adsorption Capacity Measurements

A sodium fluoride stock solution was prepared by placing 0.2210 g of sodium fluoride into 1000 mL of distilled water. A 100 mL fluoride solution with a fluoride concentration of 50 mg/L was added to a plastic-sealed conical flask for an adsorption test. Afterward, 0.05 g of adsorbent was added to a plastic-sealed conical flask containing a fluoride solution. The test solution was shaken in a thermostatic water bath shaker at 150 rpm and kept at 30°C for 24 h [30]. The effect of coexisting Cl^−^, NO_3_^−^, SO_4_^2−^, HCO_3_^−^, H_2_PO_4_^−^, Na^+^, and K^+^ ions at concentrations of 0.1, 0.4, and 0.7 mg/L was also investigated, where 0.05 g of adsorbent powder was added to 100 mL of 50 mg/L fluoride solution. The pH of the solutions was adjusted by adding 0.1 mol/L NaOH or HCl solution. The effects of pH value (2–11), initial fluoride concentration (10–100 mg/L), contact time (2–24 h), and temperature (30–50°C) on the adsorption of fluoride ion were researched, respectively. The real water sample was taken from the campus river for the fluorine removal test of the adsorbent. The adsorbent was treated with a 0.5 M NaOH solution for regeneration studies. After the adsorption equilibrium, the adsorbent was separated from the solution using a centrifuge (relative centrifugal force = 3404 *G*). The fluoride concentration was measured using an ion meter (Shanghai Instrument Electric Science Instrument Limited by Share Ltd.), and the equilibrium adsorption capacity, *q*_e_ (mg/g), of the adsorbent was calculated from the results. The *q*_e_ (mg/g) was calculated using the following equation:

(1) $$\begin{array}{c} q_{\text{e}}=\frac{\left(C_{0}-C_{\text{e}}\right)V}{m}, \end{array}$$

where *C*_0 _(mg/L) and *C*_e_ (mg/L) are the initial and equilibrium concentrations of fluoride, respectively, *V* (*L*) is the volume of the fluoride solution, and *m* (g) is the mass of the adsorbent [31].

## 3. Results and Discussion

### 3.1. Optimized Mg-Al Mixed Oxide Adsorbent

**Figure 1:**
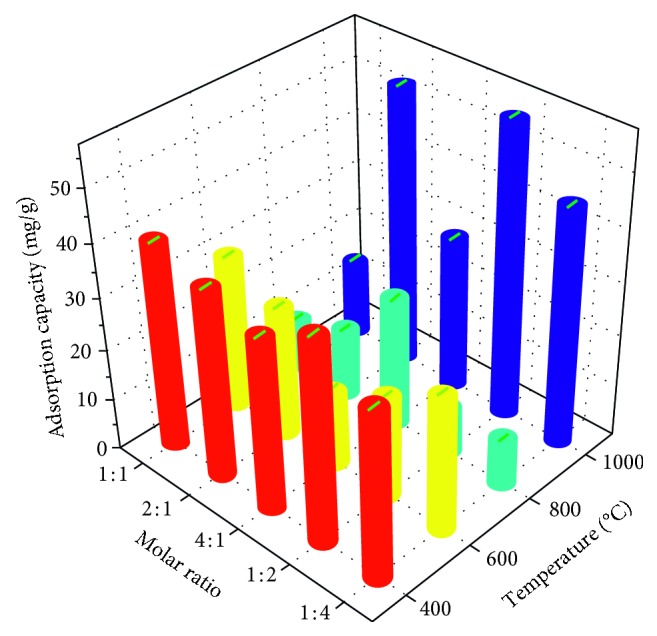
Comparison of the adsorption capacity of fluoride powders prepared using different Mg/Al molar ratios and calcination temperatures (adsorbent dose: 0.5 g/L; initial concentration of F^−^: 50 mg/L; adsorption time: 24 h; temperature: 30°C; pH: neutral conditions).

Figure 1 shows the fluoride removal ability of the Mg-Al mixed oxide adsorbents prepared using different Mg/Al molar ratios and calcination temperatures (denoted as FCT-400-I, FCT-400-II, FCT-400-III, FCT-400-IV, FCT-400-V, FCT-600-I, FCT-600-II, FCT-600-III, FCT-600-IV, FCT-600-V, FCT-800-I, FCT-800-II, FCT-800-III, FCT-800-IV, FCT-800-V, FCT-1000-I, FCT-1000-II, FCT-1000-III, FCT-1000-IV, and FCT-1000-V, respectively, where I, II, III, IV, and V represent Mg/Al molar ratios of 1 : 1, 2 : 1, 4 : 1, 1 : 2, and 1 : 4, respectively). As the calcination temperature increases, the adsorption capacity of the Mg-Al mixed oxide adsorbent powder prepared using different Mg/Al molar ratios first decreases and then increases. This behavior is attributable to the grains growing and the surface adsorption active sites covering each other as the temperature increases, followed by the crystal grains being twice crystallized, which caused the grains to be mutually displaced and the adsorption active sites to be re-exposed when the temperature was raised to 1000°C. The optimum Mg-Al mixed oxide adsorbent powder was (FCT-1000-IV) prepared using a Mg/Al molar ratio of 1 : 2 and a calcination temperature of 1000°C.

**Figure 2:**
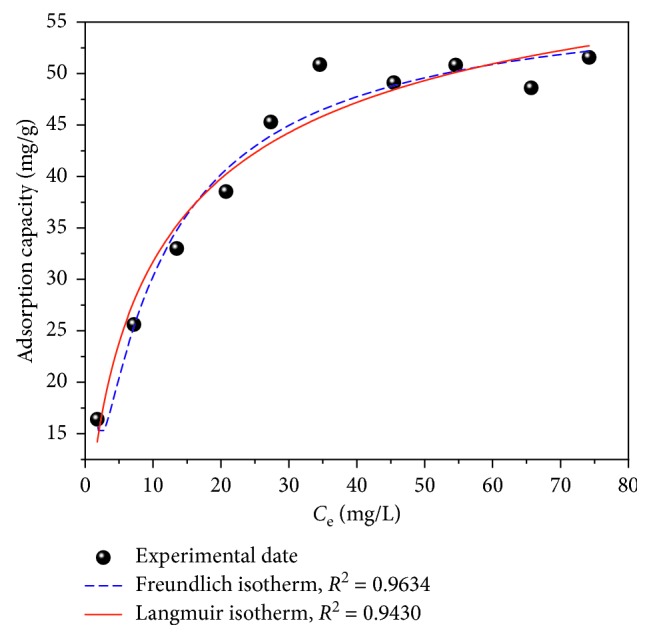
Adsorption isotherms of fluoride on FCT-1000-IV adsorbent powder (adsorbent dose: 0.5 g/L; initial concentration of F^−^: 10–100 mg/L; adsorption time: 24 h; temperature: 30°C; pH: neutral conditions).

**Figure 3:**
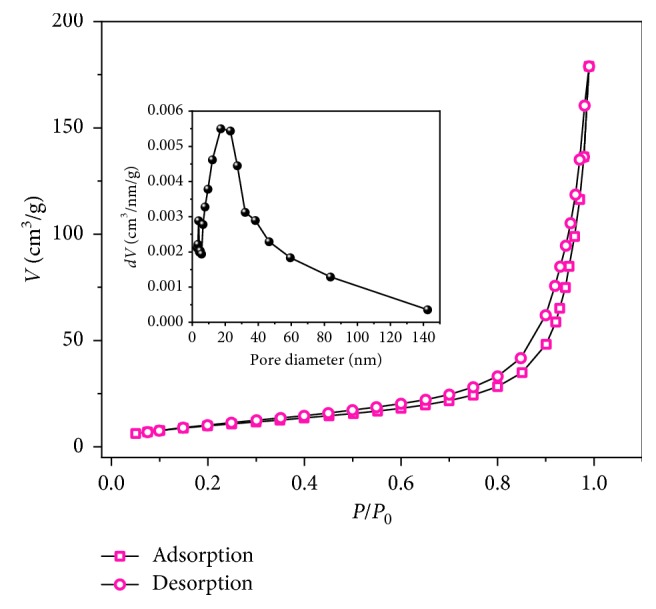
N_2_ adsorption-desorption isotherms of the FCT-1000-IV (inset: pore size distribution of the FCT-1000-IV).

The fluoride adsorption isotherm of the FCT-1000-IV Mg-Al mixed oxide adsorbent powder was studied; the results are shown in Figure 2. The maximum adsorption capacity of the FCT-1000-IV adsorbent is 53 mg/g (Figure 2). This adsorption capacity is comparable to that of the Mg/Fe-layered double hydroxide in Table 1 but is higher than the capacities of the other materials. According to the correlation coefficient (*R*^2^) given by the Langmuir and Freundlich equations, the Freundlich model is more suitable for describing the adsorption behavior, indicating that the adsorption may be due to multimolecular layer adsorption [36]. By comparison, the Mg-Al mixed oxide adsorbent prepared using a Mg/Al molar ratio of 1 : 2 and a calcination temperature of 1000°C exhibits the highest fluoride adsorption capacity. The N_2_ adsorption-desorption isotherm of the obtained product calcined at 1000°C is shown in Figure 3. The isotherm is type V with a H3 hysteresis loop, suggesting a mesoporous structure [37]. The BET surface area and average pore diameter are 45.5 m^2^/g and 28.19 nm, respectively.

**Figure 4:**
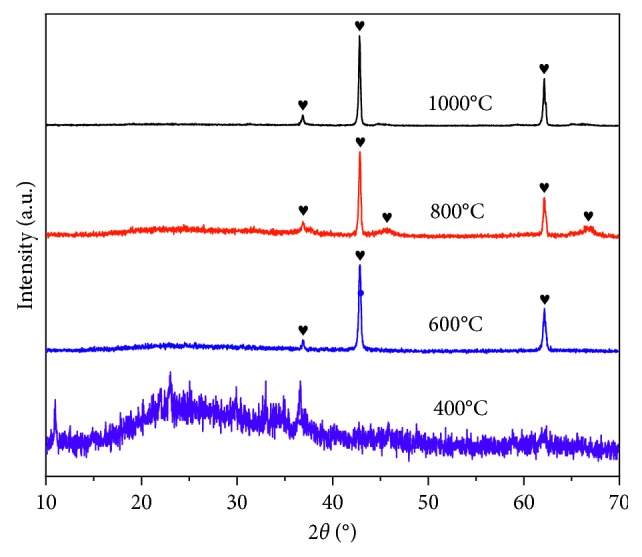
XRD patterns of the Mg-Al mixed oxide adsorbent prepared using different calcination temperatures (Mg/Al molar ratio of 1 : 2).

Figure 4 shows the XRD patterns of the Mg-Al mixed oxide adsorbent calcined at different temperatures (FCT-400-IV, FCT-600-IV, FCT-800-IV, and FCT-1000-IV). The Mg-Al mixed oxide adsorbent maintained an amorphous phase when the calcination temperature was 400°C. When the calcination temperature was increased to 600°C, the crystallization peak began to appear. Only the characteristic peaks of magnesium oxide appeared at 600°C, indicating that the alumina was still in the amorphous state. At 800°C, two characteristic peaks of magnesium oxide and aluminum oxide clearly occurred, indicating that the aluminum oxide began to crystallize; however, the peak intensity was not high and the crystallization was incomplete. After calcination at 1000°C, the characteristic peaks of aluminum oxide disappeared and were converted into aluminum-magnesium compounds. The characteristic peaks of magnesium oxide also appeared. Increasing the calcining temperature has been reported to lead to decreased adsorption capacity of the adsorbent although the maximum investigated calcination temperature in such previous studies was only 850°C [38, 39]. The literature contains no reports of an improvement in the adsorption performance after calcination at 1000°C.

**Figure 5:**
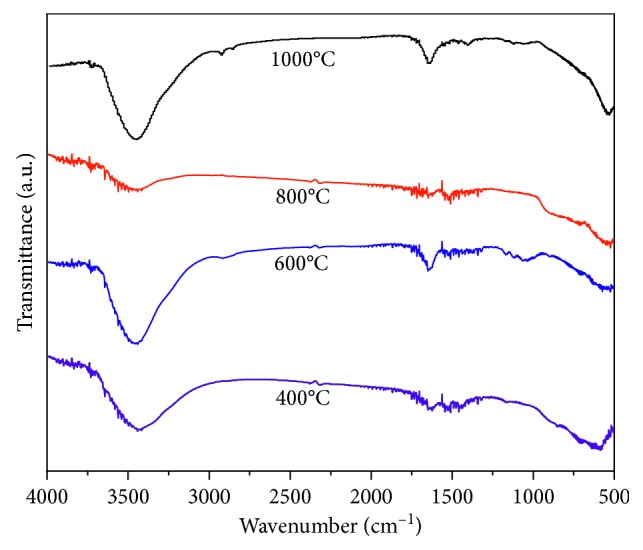
FT-IR spectra of the Mg-Al mixed oxide adsorbent using different calcination temperatures (Mg/Al molar ratio of 1 : 2).

The FT-IR spectra of the Mg-Al mixed oxide adsorbent calcined at different temperatures are shown in Figure 5 (FCT-400-IV, FCT-600-IV, FCT-800-IV, and FCT-1000-IV). As the calcining temperature increases, the peak intensity at 3466 cm^−1^ gradually decreases. However, after the calcining temperature increased to 1000°C, the peak intensity again became stronger, and two new peaks appeared at 2926 and 2855 cm^−1^. These results suggest that the Mg-Al mixed oxide calcined at 1000°C exhibits good adsorption performance because of this change. Therefore, the calcination temperature of 1000°C was used as the best adsorbent calcination temperature.

**Figure 6:**
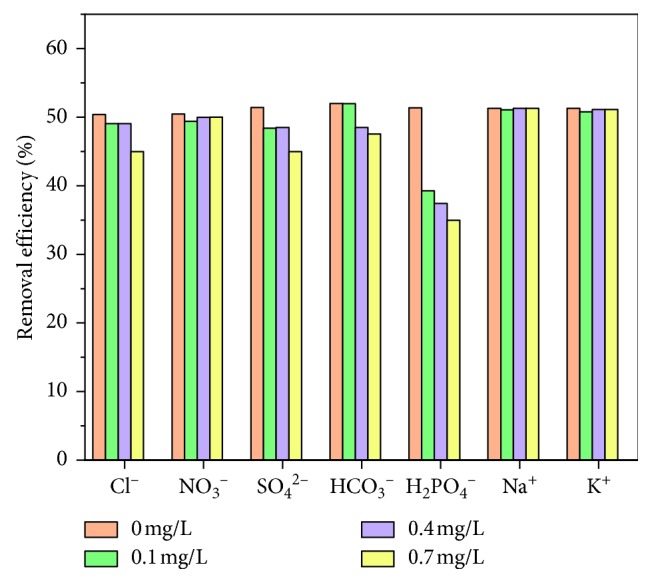
Effect of coexisting ions on fluoride removal efficiency of FCT-1000-IV (adsorbent dose: 0.5 g/L; initial concentration of F^−^: 50 mg/L; adsorption time: 24 h; temperature: 30°C; pH: neutral conditions).

**Figure 7:**
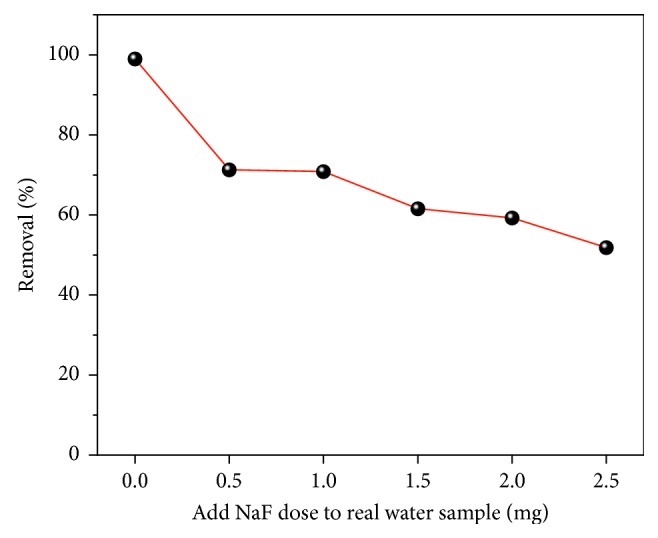
Fluoride removal from the real water samples using the FCT-1000-IV (adsorbent dose: 0.5 g/L; adsorption time: 24 h; temperature: 30°C).

The effect of coexisting ions, including Cl^−^, NO_3_^−^, SO_4_^2−^, HCO_3_^−^, H_2_PO_4_^−^, Na^+^, and K^+^ in the concentration range 0–0.7 mg/L, was evaluated using a 50 mg/L fluoride solution to test the fluoride removal efficiency of the best product (Figure 6). With increasing ion concentration of Cl^−^, NO_3_^−^, and SO_4_^2−^, Na^+^ and K^+^ have little effect on the fluoride removal efficiency. However, when the concentration of HCO_3_^−^ and H_2_PO_4_^−^ is increased, the removal efficiency of fluoride decreases from 50 to 35%. This behavior may be because the hydrolysis of HCO_3_^−^ and HPO_4_^−^ produces hydroxide ions, which increase the electrostatic repulsion in the solution, making it difficult for fluoride ions to adsorb at the adsorption sites. The results of the FCT-1000-IV defluoridation test in real water samples are shown in Figure 7. The results show that FCT-1000-IV still had good fluorine removal ability in real water samples.

### 3.2. Optimum Conditions for the Removal of Fluoride

#### 3.2.1. Effect of pH on the Adsorption Capacity

**Figure 8:**
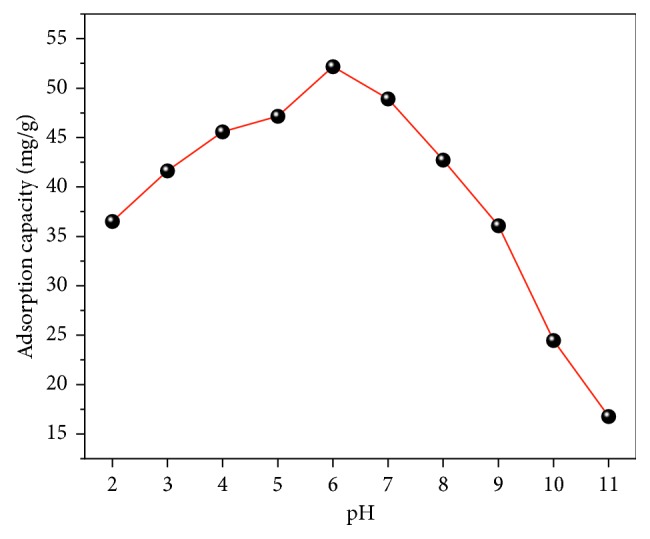
Effect of pH on the adsorption capacity of FCT-1000-IV to fluoride (adsorbent dose: 0.5 g/L; initial concentration of F^−^: 50 mg/L; adsorption time: 24 h; temperature: 30°C; 2 ≤ pH ≤ 11).

The effect of the pH value for defluoridation by the FCT-1000-IV at different pH values was investigated, as shown in Figure 8. The effect of pH on the adsorption capacity was characterized in the pH range from 2 to 11. FCT-1000-IV reached maximum adsorption capacity at pH 6. The adsorption process was more favorable under acidic conditions than under alkaline conditions; however, the neutral conditions were most suitable for the adsorption process.

#### 3.2.2. Effect of Contact Time on the Adsorption Capacity

**Figure 9:**
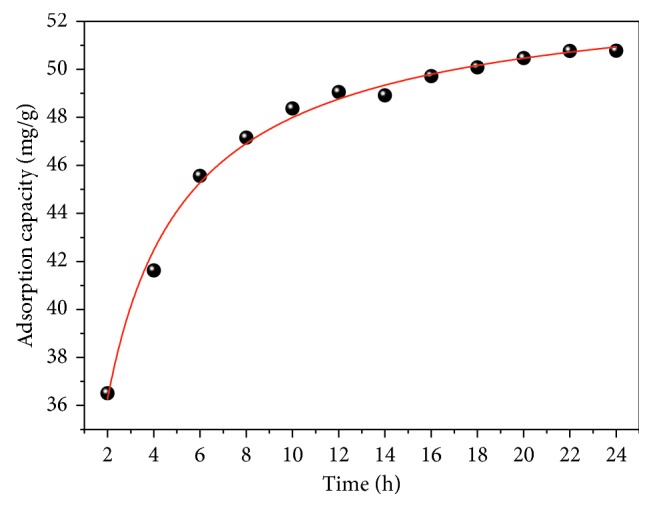
Effect of contact time on the fluoride adsorption capacity of FCT-1000-IV (adsorbent dose: 0.5 g/L; initial concentration of F^−^: 50 mg/L; adsorption time: 2–24 h; temperature: 30°C; pH: neutral conditions).

The shaking time is an important factor affecting adsorption capacity. The adsorption behavior was studied as a function of contact time from 2 to 24 h with FCT-1000-IV at 30°C (Figure 9). It is clear from the above results that the adsorption enhances with time and an equilibrium state is attained after its maximum capacity had been obtained. The fluoride removal rate increased rapidly at the beginning; however, the rate changed slowly with time beginning at 12 h. The equilibrium time was 24 h for removal of fluoride from solution by FCT-1000-IV.

#### 3.2.3. Effect of Temperature on the Adsorption Capacity

**Figure 10:**
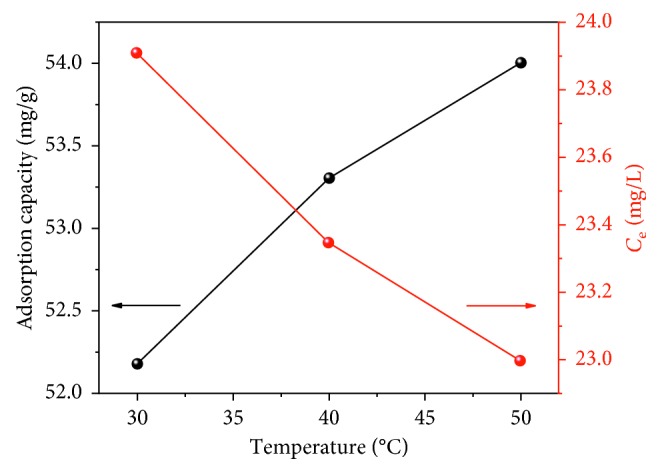
Effect of temperature on the fluoride adsorption capacity of FCT-1000-IV (adsorbent dose: 0.5 g/L; initial concentration of F^−^: 50 mg/L; adsorption time: 24 h; temperature: 30–50°C; pH: neutral conditions).

Fluoride removal was studied at different temperatures; the results are shown in Figure 10. The adsorption capacity clearly increases with increasing temperature. Thermal energy may exacerbate collisions between FCT-1000-IV particles, exposing more active sites. This effect demonstrates that temperature is one of the factors that affect the adsorption capacity. This evidence indicates that the adsorption of fluoride on FCT-1000-IV particles was an endothermic process. However, the increase in temperature had little effect on the adsorption capacity; thus, the adsorption test was still carried out at simulated room temperature.

#### 3.2.4. Effect of Initial Concentration on the Adsorption Capacity

**Figure 11:**
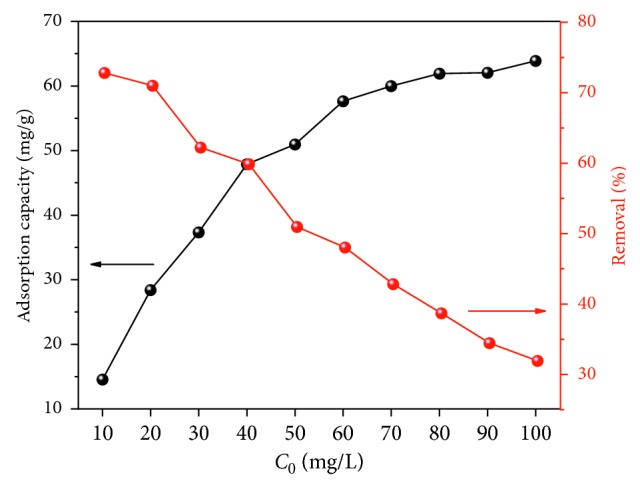
Effect of initial concentration on the fluoride adsorption capacity of FCT-1000-IV (adsorbent dose: 0.5 g/L; initial concentration of F^−^: 10–100 mg/L; adsorption time: 24 h; temperature: 30°C; pH: neutral conditions).

The effect of the initial F^−^ concentration on the F^−^ adsorption capacity and removal rate of FCT-1000-IV particles were studied with all other parameters kept constant (adsorbent dose: 0.05 g, volume of solution: 100 mL, temperature: 30°C, contact time: 24 h); the result is shown in Figure 11. In this observation, the removal rate decreased with increasing adsorption capacity. In high concentrations of fluoride solution, more active sites of the adsorbent were occupied, resulting in a reduction of the removal rate. When the *C*_0_ values reached 100 mg/L, the adsorption capacity became 63.88 mg/g and then removal rate decreased to less than 32%. At high-fluoride concentrations, the adsorption sites tend to saturate, leading to a decrease in removal rates. To ensure the high adsorption capacity of FCT-1000-IV, 0.05 g of adsorbent and a solution of 50 mg/L concentration were selected for the study.

### 3.3. Synergistic Interaction between Mg and Al

**Figure 12:**
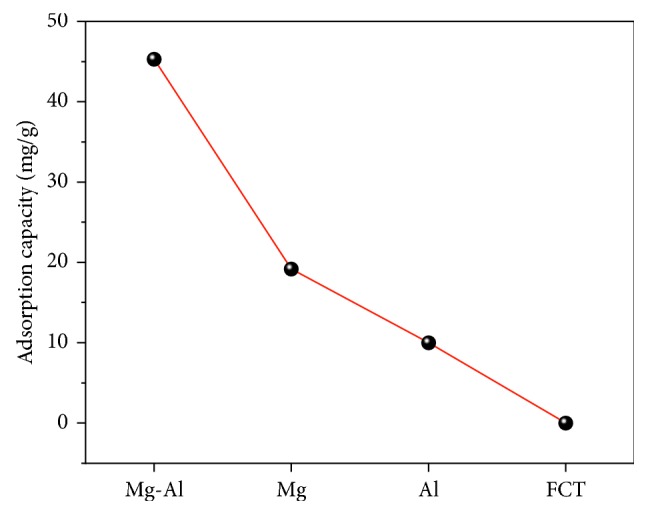
Comparison of fluoride removal ability of different media (adsorbent dose: 0.5 g/L; initial concentration of F^−^: 50 mg/L; adsorption time: 24 h; temperature: 30°C; pH: neutral conditions).

To study the synergistic interaction between Mg and Al, a Mg-Al mixed oxide adsorbent was prepared using a Mg/Al molar ratio of 1 : 2 and a calcination temperature of 1000°C. For comparison, a Mg oxide adsorbent and an Al oxide adsorbent were separately prepared under the same conditions.  Figure 12 shows the adsorption capacity of fluoride by different media. The equilibrium adsorption capacity of the Mg-Al mixed oxide adsorbent was 45.3 mg/g at an initial fluoride concentration of 50 mg/L and a sorbent dose of 0.5 g/L. The equilibrium adsorption capacities of the Mg oxide adsorbent and the Al oxide adsorbent were 19.2 mg/g and 10 mg/g, respectively. However, free FCT was not effective for removing fluoride. In addition, the equilibrium adsorption capacity of the physical mixture of the Mg oxide adsorbent and the Al oxide adsorbent with a mass ratio of Mg/Al of 1 : 1 is 17.2 mg/g, which is also lower than the equilibrium adsorbent capacity of the Mg-Al mixed oxide adsorbent.

**Figure 13:**
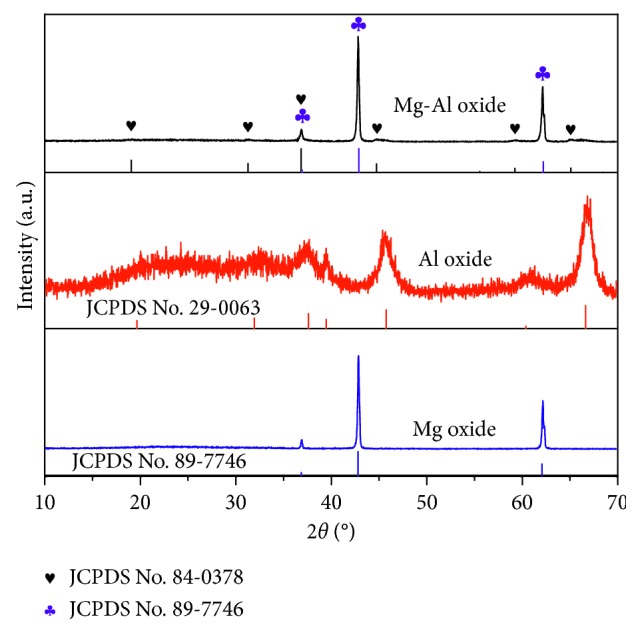
XRD patterns of the Mg oxide, Al oxide, and Mg-Al mixed oxide adsorbent calcined at 1000°C.

The XRD patterns of the Mg oxide, Al oxide, and Mg-Al mixed oxide are shown in Figure 13. The spectrum of magnesium oxide indicates the formation of face-centered cubic (FCC) magnesium oxide powder (JCPDS file No. 89-7746). The spectrum of the aluminum oxide shows that all the peaks are indexed to the cubic gamma alumina, in agreement with previous literature (JCPDS file No. 29-0063). The spectrum of the magnesium aluminum oxide shows that there are mainly two phases. One is still face-centered cubic magnesium oxide, and the other phase is newly formed Mg_0.4_Al_2.4_O_4_ (JCPDS file No. 83-0378).

**Figure 14:**
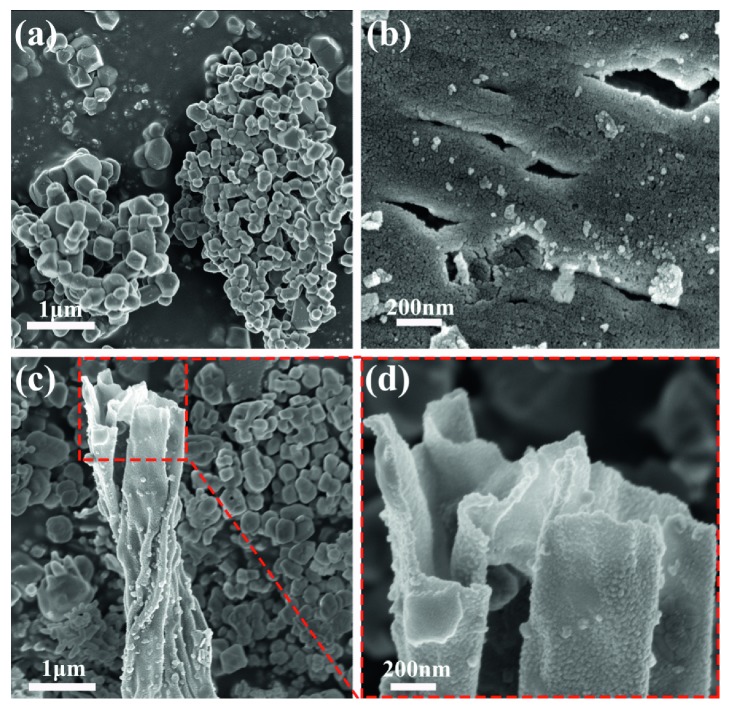
SEM images of (a) Mg oxide, (b) Al oxide, and (c, d) Mg-Al mixed oxide prepared under the same conditions.

The morphologies of the Mg oxide, Al oxide, and Mg-Al mixed oxide prepared under the same conditions are shown in Figure 14. The Mg oxide assembled into a hollow tube with 0.1 *μ*m particles (Figure 14(a)). We assumed that the removal of the FCT template after calcination caused this morphology. The morphology of the Al oxide was composed of interconnected rod-like structures with a large number of particles distributed on the surface (Figure 14(b)). The morphology of the Mg-Al mixed oxide is shown in Figure 14(c). There are two main morphologies: a 0.1 *μ*m particle similar to the Mg oxide and a flaky curly strip. A new morphology was generated while the morphology of the magnesium oxide continued to exist, which is consistent with the XRD analysis results. As shown in the partial enlargement (Figure 14(d)), small particles are still distributed on the flaky curly strip, which may be conducive to an increase in adsorption active sites.

**Figure 15:**
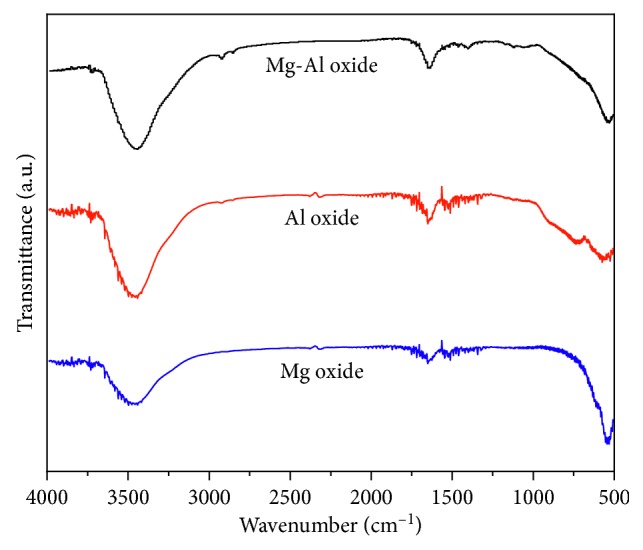
FT-IR spectra of the Mg oxide, Al oxide, and Mg-Al mixed oxide adsorbent calcined at 1000°C.

The FT-IR spectra of the Mg oxide, Al oxide, and Mg-Al mixed oxide adsorbent are shown in Figure 15. The broad band at approximately 3450 cm^−1^ is attributed to the tensile vibration of the adsorbed water, and the peak at approximately 1640 cm^−1^ is attributed to the bending vibration of the OH group [30]. The two new peaks at 1120 and 1400 cm^−1^ in the spectrum of the Mg-Al mixed oxide are attributable to the bending vibration of the hydroxyl group on the bimetallic oxide [18]. Clearly, there are no peaks in the spectrum of the Mg oxide at 2850 and 2920 cm^−1^; however, the spectrum of the Al oxide begins to show two weak peaks, and the peak intensity of the Mg-Al mixed oxide is increased.

Based on the aforementioned analysis of the adsorption capacities, XRD, morphologies, and FT-IR spectra of the adsorbent, we inferred that the Mg-Al mixed oxide adsorbent is not a simple mixture of Mg oxide and Al oxide. A synergistic interaction occurs between the Mg and Al during the synthesis.

### 3.4. Adsorption Mechanism of the Mg-Al Mixed Adsorbent

**Figure 16:**
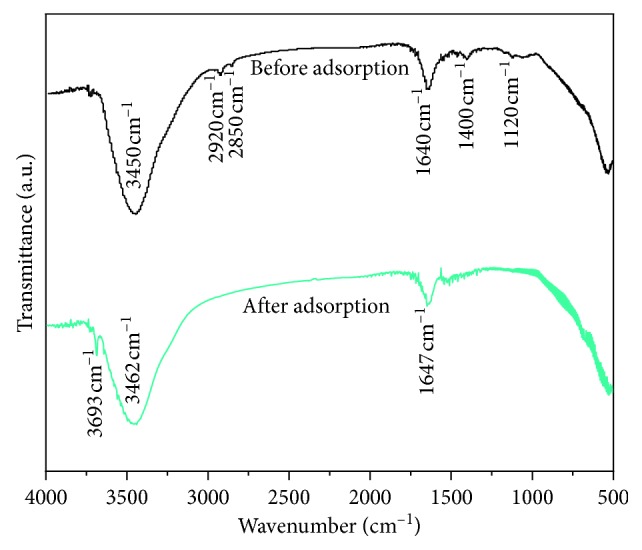
FT-IR spectra of the Mg-Al mixed oxide adsorbent before and after fluoride adsorption.

The FT-IR spectra of the Mg-Al mixed oxide adsorbent before and after fluoride adsorption are shown in Figure 16. After fluoride adsorption, the bands at 3450 and 1640 cm^−1^ representing the hydroxyl groups shifted to 3462 and 1647 cm^−1^, respectively, indicating that the fluoride interacted with the hydroxyl groups on the Mg-Al mixed oxide adsorbent. Furthermore, the peak intensity of the Mg-Al mixed oxide adsorbent at 1200, 1400, 2850, and 2920 cm^−1^ was remarkably reduced after the removal of the fluoride. The spectrum of the adsorbed adsorbent showed a sharp peak at 3690 cm^−1^ compared with the spectrum of the original adsorbent because of the vibration of the physically adsorbed water [40].

**Figure 17:**
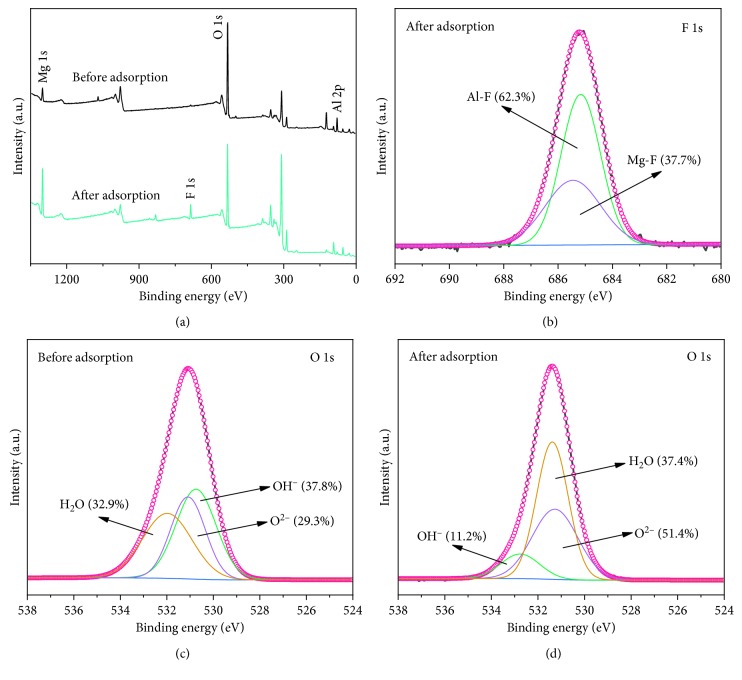
(a) XPS spectra of the Mg-Al mixed oxide adsorbent before and after fluoride adsorption; (b) F 1s spectra of the Mg-Al mixed oxide adsorbent after fluoride adsorption; (c) O 1s spectra of the Mg-Al mixed oxide adsorbent before fluoride adsorption; (d) O 1s spectra of the Mg-Al oxide adsorbent after fluoride adsorption.

To further investigate the adsorption mechanism of fluoride on the Mg-Al mixed oxide adsorbent, XPS analysis was applied to the adsorbent before and after adsorption. As shown in Figure 17(a), peaks of Al 2p, O 1s, and Mg 1s were observed for the Mg-Al oxide adsorbent before and after use. After fluoride adsorption, a new binding energy related to photoelectrons in F 1s appeared at 685.8 eV, indicating that the fluoride was adsorbed onto the Mg-Al mixed oxide adsorbent. The F 1s spectrum shown in Figure 17(b) is divided into peaks located at approximately 685.2 and 685.5 eV. The peak of 685.2 eV occupies 62.3% of the area attributed to the combination of fluorine atoms and magnesium (F-Mg) [41, 42], whereas the peak of 685.5 eV accounts for 37.7% of the area due to the combination of fluorine atoms and aluminum (F-Al) [43, 44]. The O 1s spectrum before and after fluoride adsorption is divided into three peaks: adsorbed water (H_2_O), metal oxide (O^2−^), and hydroxyl group bonded to metal (M-OH) [45]. After fluoride adsorption (Figures 17(c) and 17(d)), the surface hydroxyl groups of the adsorbent decreased from 37.8% to 11.2%. In the coordination exchange, the fluoride ion is similar in radius to the hydroxyl group, and the Mg-Al metal ion releases the hydroxyl group to form a covalent bond with fluorine. The decrease in hydroxyl groups indicates that the hydroxyl group on the surface of the Mg-Al mixed oxide adsorbent participates in the adsorption process of the fluoride, which is consistent with the results of the FT-IR analysis.

### 3.5. Reuse of Mg-Al Mixed Oxide Adsorbent

**Figure 18:**
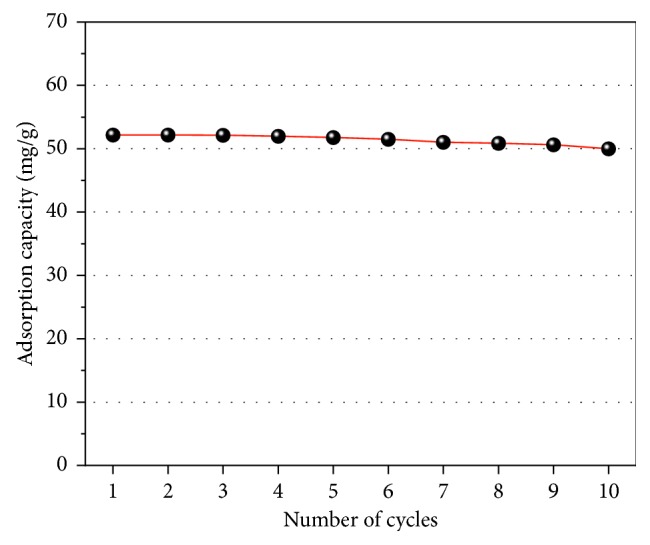
Adsorption capacity as a function of cycle number for the Mg-Al mixed oxide adsorbent.

The regeneration study shows that the alkaline environment can regenerate the discharged metal oxide adsorbent [46]. The literature includes numerous studies on the removal of fluoride by various adsorbents, but studies of the reuse of metal oxides as sorbents are rare. In the present study, we observed that the defluorination ability of FCT-1000-IV showed a downward trend after 7 cycles (Figure 18). The ability to recycle adsorbents is economically beneficial.

## 4. Conclusions

A novel Mg-Al mixed oxide adsorbent was synthesized by dipping abiological template in a magnesium chloride and aluminum chloride solution at room temperature. When the FCT was dipped into a solution with Mg/Al in a molar ratio of 1 : 2, the sample with the best adsorption performance was obtained after calcination at 1000°C. The Langmuir adsorption capacity was 53 mg/g. A synergistic interaction between Mg and Al in the Mg-Al mixed oxide adsorbent improves the fluoride adsorption capacity and leads to good resistance to interference by coexisting ions. The adsorbent exhibited ability to remove fluorine from real water samples. Good regeneration and reusability were demonstrated even after 7 cycles of use. FT-IR and XPS analyses indicated that the hydroxyl groups on the surface of the Mg-Al mixed oxide adsorbent played an important role in fluoride adsorption by ion exchange. The novel Mg-Al mixed oxide is therefore an effective and environmentally friendly adsorbent for the removal of fluoride from drinking water.

## Figures and Tables

**Table 1:** Comparison of the fluoride adsorption capacity of the Mg-Al mixed oxide adsorbent with other adsorbents.

Adsorbents	*C*_0_(mg/L)	Adsorbent dose (g/L)	*q*_e_(mg/g)	References
MgO mesoporous nanofibers	—	0.6	237.49	[32]
Mg/Fe-layered double hydroxides	—	1	50.91	[33]
Al-Ce hybrid adsorbent	2–15	0.1	27.5	[30]
Mg-Al-Zr composite	10–105	1	22.9	[31]
Amorphous aluminum hydroxide hollow spheres	5–200	1	16.77	[34]
Mg-Al-Fe compound	—	0.2	14	[35]
Mg-Al mixed oxide	10–100	0.5	53	This work

## Data Availability

The data used to support the findings of this study are available from the corresponding author upon request.
